# Patterns in Geographic Access to Health Care Facilities Across Neighborhoods in the United States Based on Data From the National Establishment Time-Series Between 2000 and 2014

**DOI:** 10.1001/jamanetworkopen.2020.5105

**Published:** 2020-05-15

**Authors:** Jennifer Tsui, Jana A. Hirsch, Felicia J. Bayer, James W. Quinn, Jesse Cahill, David Siscovick, Gina S. Lovasi

**Affiliations:** 1 Rutgers Cancer Institute of New Jersey, Rutgers, The State University of New Jersey, New Brunswick; 2 Department of Health Behavior, Society, and Policy, Rutgers School of Public Health, Rutgers, The State University of New Jersey, New Brunswick; 3 Rutgers Center for State Health Policy, Rutgers, The State University of New Jersey, New Brunswick; 4 Department of Epidemiology and Biostatistics, Dornsife School of Public Health, Drexel University, Philadelphia, Pennsylvania; 5 Urban Health Collaborative, Dornsife School of Public Health, Drexel University, Philadelphia, Pennsylvania; 6 Department of Epidemiology, Mailman School of Public Health, Columbia University, New York, New York; 7 Research, Evaluation & Policy, New York Academy of Medicine, New York, New York

## Abstract

**Importance:**

The association between proximity to health care facilities and improved disease management and population health has been documented, but little is known about small-area health care environments and how the presence of health care facilities has changed over time during recent health system and policy change.

**Objective:**

To examine geographic access to health care facilities across neighborhoods in the United States over a 15-year period.

**Design, Setting, and Participants:**

Using longitudinal business data from the National Establishment Time-Series, this cross-sectional study examined the presence of and change in ambulatory care facilities and pharmacies and drugstores in census tracts (CTs) throughout the continental United States between 2000 and 2014. Between January and April 2019, multinomial logistic regression was used to estimate associations between health care facility presence and neighborhood sociodemographic characteristics over time.

**Main Outcomes and Measures:**

Change in health care facility presence was measured as never present, lost, gained, or always present between 2000 and 2014. Neighborhood sociodemographic characteristics (ie, CTs) and their change over time were measured from US Census reports (2000 and 2010) and the American Community Survey (2008-2012).

**Results:**

Among 72 246 included CTs, the percentage of non–US-born residents, residents 75 years or older, poverty status, and population density increased, and 8.1% of CTs showed a change in the racial/ethnic composition of an area from predominantly non-Hispanic (NH) white to other racial/ethnic composition categories between 2000 and 2010. The presence of ambulatory care facilities increased from a mean (SD) of 7.7 (15.9) per CT in 2000 to 13.0 (22.9) per CT in 2014, and the presence of pharmacies and drugstores increased from a mean (SD) of 0.6 (1.0) per CT in 2000 to 0.9 (1.4) per CT in 2014. Census tracts with predominantly NH black individuals (adjusted odds ratio [aOR], 2.37; 95% CI, 2.03-2.77), Hispanic/Latino individuals (aOR 1.30; 95% CI, 1.00-1.69), and racially/ethnically mixed individuals (aOR, 1.53; 95% CI, 1.33-1.77) in 2000 had higher odds of losing health care facilities between 2000 and 2014 compared with CTs with predominantly NH white individuals, after controlling for other neighborhood characteristics. Census tracts of geographic areas with higher levels of poverty in 2000 also had higher odds of losing health care facilities between 2000 and 2014 (aOR, 1.12; 95% CI, 1.05-1.19).

**Conclusions and Relevance:**

Differential change was found in the presence of health care facilities across neighborhoods over time, indicating the need to monitor and address the spatial distribution of health care resources within the context of population health disparities.

## Key Points

**Question:**

How has change in the presence of health care facilities and pharmacies and drugstores over time across neighborhoods in the United States differed based on the race/ethnicity, age, and socioeconomic characteristics of area residents?

**Findings:**

Using business data from the National Establishment Time-Series over a 15-year period, this cross-sectional study of 72 246 census tracts found differential change in the presence of health care facilities across neighborhoods, with more disadvantaged neighborhoods never having or losing health care facilities between 2000 and 2014.

**Meaning:**

Differential geographic presence of health care resources over time can further exacerbate disparities in health care access, quality, and outcomes.

## Teaser

This cross-sectional study examines geographic access to health care facilities across neighborhoods in the United States over a 15-year period and assesses whether racial/ethnic composition and socioeconomic status are associated with change in neighborhood-level presence of health care facilities over time.

## Introduction

Geographic access to health care is associated with increased use of preventive care and improved health outcomes for certain chronic conditions.^1,2,3,4,5,6,7^ Although geographic access is one of several components that can alter an individual’s overall access to health care, including insurance status, out-of-pocket costs, facility hours, appointment wait times, and linguistic services, prior research has shown increased geographic access is associated with greater use and improved outcomes. Neighborhoods with more income inequality and residential segregation along sociodemographic lines may not attract or may underinvest in institutions that benefit the general population, resulting in unequal geographic health care access.^8^ Previous analyses of geographic access to health care services, including trauma centers, specialty care for neonatal populations, and mental health care, have indicated that neighborhoods with predominantly minority residents, lower socioeconomic status, and high residential turnover have less geographic access to care.^9,10^ This observation was confirmed by Smiley et al,^11^ who reported that health-related resources are not equally distributed across space and that disadvantage often clusters with residential racial/ethnic patterning. Although recent data indicate access to health care, as measured by insurance coverage or self-report of having a usual source of care, has improved since implementation of the Patient Protection and Affordable Care Act,^12^ few sources are available to understand geographic health care environments, including the presence of ambulatory care facilities, retail clinics, and pharmacies and drugstores, beyond county-level geographies.

Despite increasing demographic change in racial/ethnic composition and household income and aging subgroups over the last few decades, few studies have assessed temporal change in the geographic access to or the presence of health care facilities across neighborhoods in the United States. A study^13^ conducted in Illinois from 1990 to 2000 found an overall improvement in geographic access to health care over time, with worsened geographic accessibility primarily concentrated in rural areas along with a few urban pockets. Areas that experienced decreasing geographic access had higher levels of socioeconomic disadvantage, sociocultural barriers, and health care needs. Similarly, in a 2011 study, Busingye et al^14^ found substantial increases in the proportion of the population with geographic access to cardiac facilities from 1999 to 2010, with disparities still existing in rural communities. Hospital closures over the last decade and increased consolidation across hospital systems may also have altered geographic access for certain neighborhoods over time.^15,16,17,18,19^ Insights into long-term temporal trends on the availability of health care facilities nationally, particularly with respect to nonhospital facilities and attention to changing neighborhood-level sociodemographic characteristics of residents, are lacking.

This gap in the literature is addressed herein by examining change in the presence of ambulatory care facilities and pharmacies and drugstores across neighborhoods (ie, census tracts [CTs]) as a measure of geographic access in the United States over a 15-year period. Specifically, the objectives of this study were (1) to examine patterns in neighborhood-level presence of health care facilities across the United States by neighborhood-level sociodemographic characteristics and (2) to assess whether neighborhood-level population characteristics (racial/ethnic composition and socioeconomic status) were associated with change in neighborhood-level presence of health care facilities over time. We hypothesized that socioeconomically disadvantaged neighborhoods would continue to experience limited local presence of health care facilities over time compared with more advantaged neighborhoods. We also hypothesized that neighborhoods undergoing demographic compositional change across time from disadvantaged to advantaged would experience increased presence of health care facilities.

## Methods

### Study Sample

Using longitudinal business data from the National Establishment Time-Series (NETS), this cross-sectional study compiled health care environment, demographic, and socioeconomic data between 2000 and 2014 for all CTs in the continental United States (n = 72 538). Of these, 292 CTs were excluded because they contained no land area (ie, were water tracts), leaving 72 246 nonwater CTs. For consistency over time despite boundary changes, all health care environment, demographic, and socioeconomic measures were assigned to 2010 US Census geographies.

This study followed the Strengthening the Reporting of Observational Studies in Epidemiology (STROBE) reporting guideline.^20^ The study is part of a larger study (Communities Designed to Support Cardiovascular Health for Older Adults^52^) that includes human participants not included in this analysis and was approved by the Drexel University Institutional Review Board.

### Dependent Variable of Health Care Facilities

To characterize neighborhood-level geographic access, the presence of health care facilities was measured using 2000 to 2014 business data from the NETS database, licensed from Walls & Associates (Denver, Colorado) in January 2017. Detailed methods on the creation and cleaning of the NETS data can be found elsewhere.^21,22^ Briefly, the NETS pulls annual snapshots of Dun & Bradstreet (Short Hills, New Jersey) business data to create time series information on all names of US businesses, years active, and industrial classification using Standard Industrial Classification (SIC) codes. The NETS data represent a census of all businesses across the United States, and the NETS is considered one of the most comprehensive databases of establishments available. Prior studies^2,23,24^ have used the NETS data to examine health care facilities and specific chronic conditions. From the NETS, records were categorized as ambulatory care facilities or as pharmacies and drugstores using SIC codes (eTable 1 in the Supplement). Ambulatory care was a category designed to capture locations able to provide outpatient care, including screenings and other preventive measures. As such, ambulatory care captures offices or clinics of health practitioners, mental health outpatient and continuous care facilities, behavioral health outpatient and continuous care facilities, urgent care locations, retail clinics, physical therapists, kidney centers, and dental care facilities. The pharmacy and drugstore category was designed to capture locations where medications and medical supplies could be purchased. To capture national chain pharmacies and drugstores otherwise missed because of incorrect SIC code, we searched a broader set of SIC codes for any company or trade name that was on the Nielsen (New York, New York) TDLinx list for trade channel “drug” and subchannels “conventional drug store” or “Rx only and small drug store.”

Health care facilities were geocoded and aggregated to CT for each year in which a business was open, focused on 2000 and 2014 only. Using this information, counts of health care facilities per CT were calculated. After examining the distribution of counts, CTs were classified as having none vs any for each type of health care facility (ambulatory care facilities or pharmacies and drugstores). Census tracts were divided into the following 4 trajectories of health care facility presence over time between 2000 and 2014: (1) never having any facilities (CTs having none at both time points), (2) losing (CTs going from having at least 1 to having none), (3) gaining (CTs going from having none to having at least 1), and (4) always having a facility (CTs having at least 1 at both time points).

### Independent Variables of Demographic and Socioeconomic Characteristics

Neighborhood demographic and socioeconomic characteristics for 2000 and 2010 were accessed using the Longitudinal Tract Database.^25,26^ This database harmonizes data from US Census reports (2000 and 2010) and the American Community Survey (2008-2012), accounting for differences in geographies and measurements over time. Demographic and socioeconomic characteristics were selected to represent a range of domains while minimizing collinearity. To classify neighborhood demographic characteristics, we used the proportion of residents identifying as non-Hispanic (NH) white individuals, NH black individuals, Hispanic/Latino individuals, NH Asian/Pacific Islander individuals, and non–US born individuals, and those aged 75 years or older. Racial/ethnic composition of neighborhoods was assessed by predominant (>60%) racial/ethnic group into the following racial/ethnic categories: predominantly NH white, predominantly NH black, predominantly Hispanic/Latino, or predominantly NH Asian/Pacific Islander. Places with no predominant group were classified as racially/ethnically mixed areas. These categorizations were based on prior use in the literature.^27^ To represent socioeconomic conditions, we used the proportion of residents living at 100% of the federal poverty level, the proportion with a high school (HS) diploma or less, and home ownership.

For linear variables, change between 2000 and 2010 was calculated by subtracting 2000 values from 2010 values for each CT. For racial/ethnic composition, places that had the same racial/ethnic composition at both periods were classified as remaining predominantly NH white, NH black, Hispanic/Latino, NH Asian/Pacific Islander, or racially/ethnically mixed areas. Change in the racial/ethnic composition of an area was classified in the following 3 ways: (1) a change from predominantly NH white to predominantly NH black, predominantly Hispanic/Latino, predominantly NH Asian/Pacific Islander, or racially/ethnically mixed; (2) a change to predominantly NH white from predominantly NH black, predominantly Hispanic/Latino, predominantly NH Asian/Pacific Islander, or racially/ethnically mixed; and (3) all other changes.

### Statistical Analysis

Between January and April 2019, we calculated descriptive statistics of health care facilities, demographics, and socioeconomic characteristics for 2000, 2010, and 2014 (health care facilities only). Categories of health care facility presence over time were mapped, and frequencies were compared across states. Bivariate analyses were conducted using both baseline (2000) and change (2000-2010) in demographic and socioeconomic characteristics across categories to predict change in health care facilities between 2000 and 2014. Because bivariate analyses include all nonwater CTs in the continental United States (rather than a sample), multinomial logistic regression was used to estimate associations between initial (2000) demographic and socioeconomic characteristics and change in health care facility (2000-2014). Longitudinal multinomial logistic regression models estimated associations between change (2000-2010) in demographic and socioeconomic characteristics and change in health care facility presence (2000-2014). These models were selected instead of a multilevel logistic regression because the intraclass correlation coefficients for outcomes within states were all low (1.4%-6.4%).^28^ Models were mutually adjusted for all predictors; longitudinal models were also adjusted for living at 100% of the federal poverty level, the proportion of residents with a HS diploma or less, and baseline (2000) population density (population per square kilometer). Select results for specific categories of health care facility change in CTs of areas with predominantly NH Asian/Pacific Islander residents are not given because of small sample sizes of CTs (n <10).

## Results

### Description of CTs

In 2000, most CTs included individuals who were predominantly NH white, with racially/ethnically mixed individuals being second most common, followed by predominantly NH black individuals (Table 1). Between 2000 and 2010, 8.1% of CTs showed a change in the racial/ethnic composition of an area from predominantly NH white to one of the other racial/ethnic composition categories, 0.9% showed a change to predominantly NH white from one of the other categories, and 3.9% showed a change between the other categories (Table 2). Census tract proportion of non–US-born residents, those 75 years or older, those living below poverty level, and the population density increased between 2000 and 2010. In contrast, the proportion of residents with a HS diploma or less and those who owned a home decreased.

**Table 1.** Demographic and Socioeconomic Characteristics (2000) Across Categories of Change in Health Care Facilities (2000-2014) for 72 246 Continental Nonwater US Census Tracts

2000 Characteristic^a^	No. (%)								
	All census tracts (N = 72 246)	Ambulatory care facilities				Pharmacies and drugstores			
		None (n = 6035)	Lose (n = 2020)	Gain (n = 10 644)	Always (n = 53 547)	None (n = 32 223)	Lose (n = 5650)	Gain (n = 11 563)	Always (n = 22 810)
Race/ethnicity^b^									
Predominantly NH white	51 329 (71.1)	3289 (54.5)	1110 (55.0)	7583 (71.2)	39 347 (73.5)	22 275 (69.1)	3902 (69.1)	8500 (73.5)	16 652 (73.0)
Predominantly NH black	4946 (6.9)	902 (15.0)	373 (18.5)	695 (6.5)	2976 (5.6)	2545 (7.9)	530 (9.4)	602 (5.2)	1269 (5.6)
Predominantly Hispanic/Latino	3453 (4.8)	470 (7.8)	136 (6.7)	573 (5.4)	2274 (4.3)	1691 (5.3)	235 (4.2)	522 (4.5)	1005 (4.4)
Predominantly NH Asian/Pacific Islander	120 (0.2)	6 (0.1)	1 (0.1)	17 (0.2)	96 (0.2)	43 (0.1)	8 (0.1)	21 (0.2)	48 (0.2)
Racially/ethnically mixed	12 398 (17.2)	1368 (22.7)	400 (19.8)	1776 (16.7)	8854 (16.5)	5669 (17.6)	975 (17.3)	1918 (16.6)	3836 (16.8)
Non–US born, mean (SD), %	10.4 (13.2)	8.9 (13.2)	8.9 (13.1)	9.2 (12.1)	10.9 (13.3)	9.8 (12.7)	9.6 (12.1)	11.3 (13.1)	11.0 (14.1)
Aged ≥75 y, mean (SD), %	6.0 (4.4)	4.8 (3.8)	5.5 (3.9)	4.7 (4.1)	6.5 (4.5)	5.4 (4.2)	6.5 (4.1)	5.5 (4.6)	7.2 (4.5)
Living below poverty, mean (SD), %	12.8 (11.1)	17.8 (13.5)	18.5 (13.2)	11.9 (11.0)	12.2 (10.5)	13.1 (11.6)	13.8 (11.2)	10.6 (10.1)	13.4 (10.5)
HS diploma or less, mean (SD), %	48.6 (19.2)	60.6 (17.7)	60.2 (15.7)	49.8 (18.7)	46.9 (18.7)	50.0 (19.4)	50.1 (18.2)	43.6 (18.8)	49.6 (18.2)
Home ownership, mean (SD), %	66.1 (23.3)	65.3 (25.6)	63.7 (23.3)	73.2 (22.0)	65.2 (22.5)	68.5 (23.3)	63.6 (21.9)	69.1 (23.1)	62.5 (22.0)
Population per km^2^, mean (SD)	1984.8 (4552.5)	1832.4 (4263.7)	2021.1 (3846.2)	1379.5 (3567.1)	2120.9 (4767.9)	1766.4 (3861.1)	1914.4 (3701.7)	1908.4 (4283.3)	2349.5 (5629.1)

Abbreviations: HS, high school; NH, non-Hispanic.

^a^ Data are from the 2000 US Census report unless otherwise indicated.

^b^ Racial/ethnic composition was assessed by predominant (>60%) racial/ethnic group. Places with no predominant group were classified as racially/ethnically mixed areas.

**Table 2.** Change in Demographic and Socioeconomic Characteristics (2000-2010) Across Categories of Change in Health Care Facilities (2000-2014) for 72 246 Continental Nonwater US Census Tracts

Change in characteristic (2000-2010)	No. (%)								
	All census tracts (N = 72 246)	Ambulatory care facilities				Pharmacies and drugstores			
		None (n = 6035)	Lose (n = 2020)	Gain (n = 10 644)	Always (n = 53 547)	None (n = 32 223)	Lose (n = 5650)	Gain (n = 11 563)	Always (n = 22 810)
Race/ethnicity^a^^,^^b^									
Remained predominantly NH white	45 513 (63.0)	2878 (47.7)	972 (48.1)	6636 (62.3)	35 027 (65.4)	19 906 (61.8)	3439 (60.9)	7348 (63.6)	14 820 (65.0)
Remained predominantly NH black	4421 (6.1)	816 (13.5)	341 (16.9)	619 (5.8)	2645 (4.9)	2276 (7.1)	480 (8.5)	534 (4.6)	1131 (5.0)
Remained predominantly Hispanic/Latino	3267 (4.5)	447 (7.4)	126 (6.2)	537 (5.1)	2157 (4.0)	1596 (5.0)	218 (3.9)	496 (4.3)	957 (4.2)
Remained predominantly NH Asian/Pacific Islander	111 (0.2)	4 (0.1)	1 (0.1)	15 (0.1)	91 (0.2)	37 (0.1)	8 (0.1)	20 (0.2)	46 (0.2)
Remained racially/ethnically mixed	9660 (13.4)	1016 (16.8)	298 (14.8)	1336 (12.6)	7010 (13.1)	4358 (13.5)	760 (13.5)	1507 (13.0)	3035 (13.3)
Changed from predominantly NH white	5816 (8.1)	411 (6.8)	138 (6.8)	947 (8.9)	4320 (8.1)	2369 (7.4)	8.2 (463)	1152 (10.0)	1832 (8.0)
Changed to predominantly NH white	645 (0.9)	92 (1.5)	16 (0.8)	128 (1.2)	409 (0.8)	347 (1.1)	31 (0.6)	116 (1.0)	151 (0.7)
All other changes	2813 (3.9)	371 (6.2)	128 (6.3)	426 (4.0)	1888 (3.5)	1334 (4.1)	251 (4.4)	390 (3.4)	838 (3.7)
Non–US born, mean (SD), %^c^	1.7 (5.4)	1.3 (6.7)	1.2 (6.6)	2.1 (5.9)	1.7 (5.1)	1.5 (5.6)	1.6 (5.0)	2.3 (5.7)	1.7 (4.9)
Aged ≥75 y, mean (SD), %^b^	0.3 (2.6)	0.2 (3.5)	0.1 (2.3)	0.5 (2.7)	0.2 (2.5)	0.4 (2.8)	0.0 (2.3)	0.4 (2.7)	0.1 (2.4)
Living below poverty, mean (SD), %^c^	3.1 (7.6)	3.0 (11.4)	3.9 (9.6)	2.5 (7.9)	3.1 (6.9)	2.9 (8.4)	3.7 (7.5)	2.8 (7.0)	3.3 (6.9)
HS diploma or less, mean (SD), %^c^	−5.2 (8.5)	−6.3 (13.0)	−5.7 (9.8)	−5.9 (9.5)	−5.0 (7.5)	−5.5 (9.4)	−5.0 (7.8)	−4.9 (8.4)	−5.2 (7.2)
Home ownership, mean (SD), %^b^	−2.2 (8.5)^c^	−3.4 (14.0)	−2.2 (8.6)	−2.2 (10.3)	−2.1 (7.2)	−2.0 (9.7)	−2.3 (7.0)	−2.6 (9.3)	−2.4 (6.5)
Population per km^2^, mean (SD)^b^	33.7 (705.6)	−44.3 (791.9)	−72.7 (666.8)	115.7 (835.0)	28.9 (665.8)	18.7 (647.9)	−27.3 (537.9)	108.8 (807.0)	28.9 (760.4)

Abbreviations: HS, high school; NH, non-Hispanic.

^a^ Racial/ethnic composition assessed by predominant (>60%) racial/ethnic group. Places with no predominant group were classified as racially/ethnically mixed areas. For 2000 to 2010, census tracts that remained predominantly one race/ethnicity were grouped in their own category. Change in racial/ethnic composition was classified in the following 3 ways: (1) a change from predominantly NH white to predominantly NH black, predominantly Hispanic/Latino, predominantly NH Asian/Pacific Islander, or racially/ethnically mixed; (2) a change to predominantly NH white from predominantly NH black, predominantly Hispanic/Latino, predominantly NH Asian/Pacific Islander, or racially/ethnically mixed; and (3) all other changes.

^b^ Second period data (ie, change from baseline [2000] to 2010) are from the 2010 decennial census.

^c^ Second period data estimates are from the American Community Survey (2008-2012).

### Description of Change in Health Care Facilities

Census tracts had many more ambulatory care facilities than pharmacies and drugstores, and both facility types increased between 2000 and 2014. The mean (SD) count of pharmacies and drugstores was less than 1 per CT (0.6 [1.0] in 2000 and 0.9 [1.4] in 2014, respectively). Conversely, for ambulatory care facilities, CTs had a mean (SD) of 7.7 (15.9) in 2000 and 13.0 (22.9) in 2014.

For most CTs, the presence or absence was stable over time, with 8.4% never having ambulatory care facilities, 74.1% always having at least one ambulatory care facility, 44.6% never having pharmacies and drugstores, and 31.6% always having a pharmacy (Table 1). However, a substantial percentage of CTs went from having no facilities in 2000 to having at least one by 2014 (14.7% for ambulatory care facilities and 16.0% for pharmacies and drugstores) . A smaller percentage went from having at least one facility to having no facilities (2.8% for ambulatory care facilities and 7.8% for pharmacies and drugstores). Mapping these categories revealed no clear regional pattern across the United States (Figure), although substantial differences by state existed over time (eFigure 1, eFigure 2, eFigure 3, and eFigure 4 in the Supplement). For example, a higher proportion of CTs within states in the Northeast (ie, Massachusetts, New Jersey, Connecticut, Pennsylvania, and Rhode Island) had consistent availability of health care facilities, and a higher proportion of CTs within states in the South and Southwest (ie, Nevada, New Mexico, and Alabama) had none of these health care facilities in either 2000 or 2014.

**Figure.**
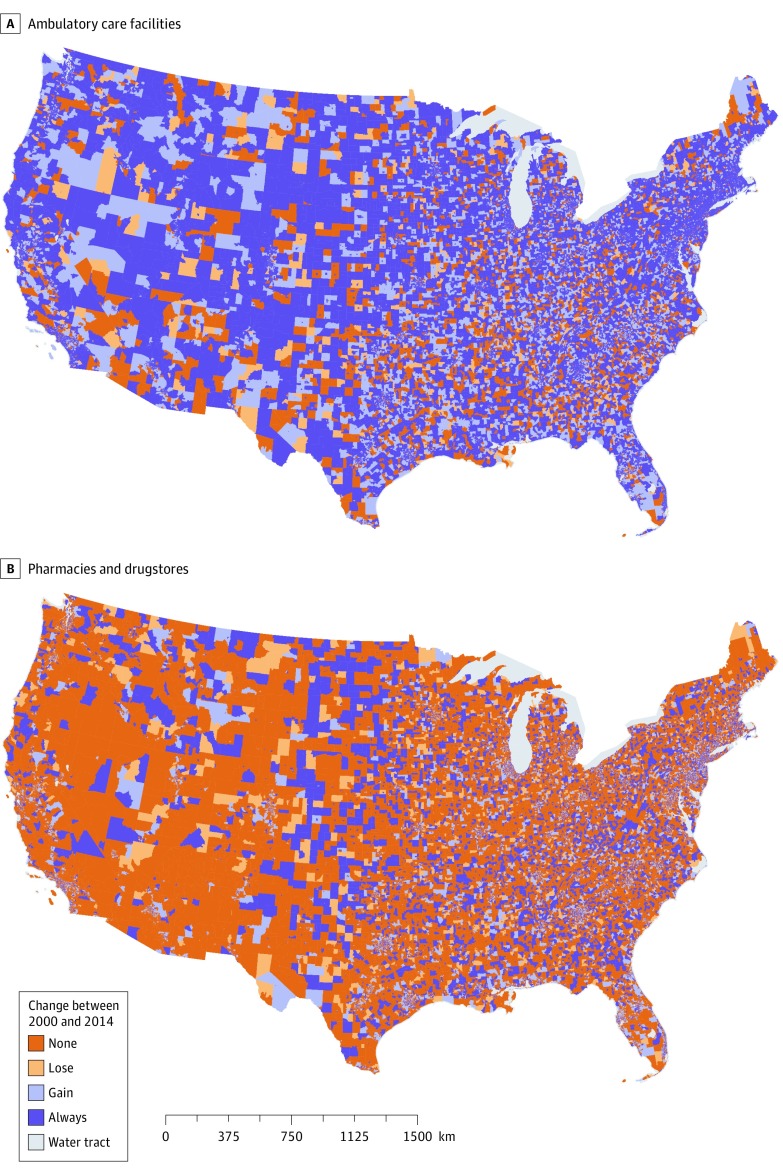
Change in the Presence of Health Care Facilities Between 2000 and 2014 A and B, Census tracts were divided into the following 4 trajectories of health care facility presence over time between 2000 and 2014: never having any facilities, losing, gaining, and always having a facility. Mapping these categories revealed no clear regional pattern across the United States.

Results from the bivariate analyses indicated that CTs for areas that gained or consistently had health care facilities were more likely in 2000 to have a racial/ethnic composition that was predominantly NH white or predominantly NH Asian/Pacific Islander, have a higher proportion of non–US-born residents, have a lower proportion of residents living below poverty level, and have a lower proportion of residents with a HS diploma or less (Table 1). Across categories of health care facility presence over time, similar patterns emerged for change in demographic and socioeconomic characteristics between 2000 and 2010 (Table 2) and for 2010 (eTable 2 in the Supplement).

### Characteristics in 2000 Associated With Change in Health Care Facilities Between 2000 and 2014

Consistent with our hypotheses about demographic characteristics, CTs of areas with a racial/ethnic composition classified as predominantly NH black, predominantly Hispanic/Latino, or racially/ethnically mixed in 2000 were more likely to never have any or to lose ambulatory care facilities between 2000 and 2014 than predominantly NH white tracts (Table 3). Census tracts of areas with a racial/ethnic composition classified in 2000 as predominantly NH black (adjusted odds ratio [aOR], 2.00; 95% CI, 1.81-2.22), predominantly Hispanic/Latino (aOR, 1.67; 95% CI, 1.42-1.96), and racially/ethnically mixed (aOR, 1.84; 95% CI, 1.69-2.00) had higher odds of never having any ambulatory care facilities compared with areas classified as predominantly NH white. Similarly, after controlling for other neighborhood characteristics, CTs of areas classified in 2000 as predominantly NH black (aOR, 2.37; 95% CI, 2.03-2.77), predominantly Hispanic/Latino (aOR, 1.30; 95% CI, 1.00-1.69), and racially/ethnically mixed (aOR, 1.53; 95% CI, 1.33-1.77) had higher odds of losing ambulatory care facilities than CTs of areas classified as predominantly NH white. In terms of gaining ambulatory care facilities, CTs of areas with a racial/ethnic composition classified in 2000 as predominantly NH black (aOR, 1.30; 95% CI, 1.18-1.44), predominantly Hispanic/Latino (aOR, 1.26; 95% CI, 1.10-1.45), and racially/ethnically mixed (aOR, 1.26; 95% CI, 1.17-1.36) had substantial odds of gaining ambulatory care facilities compared with CTs of areas classified as predominantly NH white. Conversely, CTs with a higher proportion of non–US-born residents or individuals 75 years or older were less likely to never have any health care facilities, to have lost facilities, or to have gained facilities. Similar patterns, albeit slightly less pronounced, emerged for change in the presence of pharmacies and drugstores between 2000 and 2014.

**Table 3.** Change in Health Care Facilities (2000-2014) by Demographic and Socioeconomic Characteristics (2000) Using Multinomial Logistic Regression for 72 246 Continental Nonwater US Census Tracts

2000 Characteristic^a^	aOR (95% CI)^b^					
	Ambulatory care facilities change			Pharmacies and drugstores change		
	None vs always	Lose vs always	Gain vs always	None vs always	Lose vs always	Gain vs always
Race/ethnicity^c^						
Predominantly NH black vs NH white	2.00 (1.81-2.22)	2.37 (2.03-2.77)	1.30 (1.18-1.44)	1.72 (1.59-1.87)	1.91 (1.68-2.16)	1.62 (1.45-1.82)
Predominantly Hispanic/Latino vs NH white	1.67 (1.42-1.96)	1.30 (1.00-1.69)	1.26 (1.10-1.45)	1.48 (1.32-1.66)	1.40 (1.16-1.70)	1.44 (1.24-1.67)
Predominantly NH Asian/Pacific Islander vs NH white	Not listed^d^	Not listed^d^	1.60 (0.92-2.76)	1.03 (0.66-1.61)	Not listed^d^	0.64 (0.37-1.12)
Racially/ethnically mixed vs NH white	1.84 (1.69-2.00)	1.53 (1.33-1.77)	1.26 (1.17-1.36)	1.33 (1.26-1.41)	1.27 (1.15-1.40)	1.18 (1.09-1.28)
Non–US born, %	0.67 (0.64-0.70)	0.77 (0.71-0.82)	0.90 (0.87-0.93)	0.95 (0.92-0.97)	0.90 (0.86-0.94)	1.14 (1.10-1.18)
Aged ≥75 y, %	0.45 (0.43-0.47)	0.69 (0.65-0.74)	0.48 (0.47-0.50)	0.63 (0.62-0.64)	0.89 (0.87-0.92)	0.69 (0.67-0.71)
Living below poverty, %	1.02 (0.98-1.07)	1.12 (1.05-1.19)	1.08 (1.04-1.12)	1.11 (1.08-1.14)	1.00 (0.96-1.05)	0.99 (0.95-1.03)
HS diploma or less, %	2.35 (2.25-2.45)	2.06 (1.93-2.21)	1.28 (1.25-1.32)	0.98 (0.96-1.00)	0.99 (0.95-1.02)	0.74 (0.71-0.76)
Home ownership, %	1.25 (1.20-1.30)	1.22 (1.13-1.31)	1.69 (1.63-1.75)	1.52 (1.48-1.56)	1.06 (1.02-1.11)	1.46 (1.41-1.51)
Population per km^2^	0.99 (0.96-1.03)	1.00 (0.95-1.06)	0.98 (0.94-1.01)	0.99 (0.97-1.01)	0.94 (0.90-0.97)	0.97 (0.95-1.00)

Abbreviations: aOR, adjusted odds ratio; HS, high school; NH, non-Hispanic.

^a^ All linear variables are standardized such that estimates are equal to a 1-SD increase from the mean.

^b^ Models mutually adjusted for all other characteristics in 2000.

^c^ Racial/ethnic composition was assessed by predominant (>60%) racial/ethnic group. Places with no predominant group were classified as racially/ethnically mixed areas.

^d^ Results for predominantly NH Asian/Pacific Islander census tracts that lost or had no health care facilities are suppressed because of small sample size (n <10).

Results for socioeconomic variables were more mixed (Table 3). Consistent with our hypotheses, CTs in 2000 that included areas with a high proportion of individuals living below poverty level (aOR, 1.12; 95% CI, 1.05-1.19) and high proportion of individuals with a HS diploma or less (aOR, 2.06; 95% CI, 1.93-2.21) were more likely to have lost ambulatory care facilities. However, areas with a higher proportion of home ownership in 2000 was similarly associated with losing ambulatory care facilities (aOR, 1.22; 95% CI, 1.13-01.31). Only home ownership in 2000 was associated with an increased odds of losing pharmacies and drugstores between 2000 and 2014 (aOR, 1.06; 95% CI, 1.02-1.11).

### Change in Characteristics Between 2000 and 2010 Associated With Change in Health Care Facilities Between 2000 and 2014

In general, CTs of areas with a racial/ethnic composition that remained predominantly NH black, predominantly Hispanic/Latino, and racially/ethnically mixed between 2000 and 2010 were more likely to never have any or to lose health care facilities between 2000 and 2014 than CTs of areas with a composition that remained predominantly NH white (Table 4). Census tracts of areas with a racial/ethnic composition that remained predominantly NH black had 251% (aOR, 2.51; 95% CI, 2.25-2.80) higher odds of having no ambulatory care facilities and 202% (aOR, 2.02; 95% CI, 1.86-2.20) higher odds of having no pharmacies or drugstores in both 2000 and 2014 compared with CTs of areas with a composition that remained NH white. Similarly, CTs of areas with a racial/ethnic composition that remained predominantly NH black had 260% (aOR, 2.60; 95% CI, 2.20-3.07) and 199% (aOR, 1.99; 95% CI, 1.75-2.27) higher odds of losing their ambulatory care facility and pharmacy or drugstore between 2000 and 2014, respectively compared with CTs of areas with a racial/ethnic composition that remained NH white. Notably, CTs of areas in which the racial/ethnic composition changed to predominantly NH white had 345% (aOR, 3.45; 95% CI, 2.70-4.41) higher odds of never having an ambulatory care facility and 184% (aOR, 1.84; 95% CI, 1.51-2.24) higher odds of never having a pharmacy or drugstore between 2000 and 2014. Places that experienced increases in proportion of non–US-born residents or elderly individuals 75 years or older between 2000 and 2010 were more likely to gain (vs always having) health care facilities between 2000 and 2014.

**Table 4.** Change in Health Care Facilities (2000-2014) by Change in Demographic and Socioeconomic Characteristics (2000-2010) Using Multinomial Logistic Regression for 72 246 Continental Nonwater US Census Tracts

Change in characteristic (2000-2010)^a^	aOR (95% CI)					
	Ambulatory care facilities change^b^			Pharmacies and drugstores change^b^		
	None vs always	Lose vs always	Gain vs always	None vs always	Lose vs always	Gain vs always
Race/ethnicity^c^						
Remained predominantly NH black	2.51 (2.25-2.80)	2.60 (2.20-3.07)	1.92 (1.73-2.14)	2.02 (1.86-2.20)	1.99 (1.75-2.27)	1.97 (1.75-2.22)
Remained predominantly Hispanic/Latino	1.10 (0.96-1.25)	0.80 (0.64-1.00)	1.76 (1.57-1.97)	1.72 (1.57-1.90)	1.19 (1.01-1.41)	2.66 (2.34-3.03)
Remained predominantly NH Asian/Pacific Islander	Not listed^d^	Not listed^d^	1.44 (0.83-2.51)	0.81 (0.52-1.26)	Not listed^d^	1.12 (0.65-1.92)
Remained racially/ethnically mixed	1.64 (1.50-1.78)	1.27 (1.09-1.46)	1.29 (1.20-1.38)	1.33 (1.26-1.41)	1.19 (1.08-1.31)	1.41 (1.31-1.51)
Changed from predominantly NH white	1.35 (1.20-1.51)	1.30 (1.08-1.58)	1.30 (1.20-1.41)	1.13 (1.06-1.21)	1.15 (1.03-1.29)	1.31 (1.21-1.43)
Changed to predominantly NH white	3.45 (2.70-4.41)	1.54 (0.92-2.57)	1.92 (1.56-2.37)	1.84 (1.51-2.24)	0.96 (0.65-1.43)	1.84 (1.43-2.37)
All other changes	1.71 (1.51-1.95)	1.57 (1.28-1.92)	1.49 (1.32-1.67)	1.55 (1.41-1.71)	1.47 (1.26-1.72)	1.57 (1.38-1.79)
Non–US born, %	0.98 (0.95-1.00)	0.95 (0.90-0.99)	1.07 (1.05-1.10)	0.98 (0.97-1.00)	0.98 (0.95-1.01)	1.09 (1.07-1.12)
Aged ≥75 y, %	1.12 (1.08-1.15)	1.05 (1.00-1.11)	1.11 (1.09-1.14)	1.13 (1.10-1.15)	0.98 (0.95-1.01)	1.12 (1.09-1.15)
Living below poverty, %	0.91 (0.88-0.93)	1.02 (0.98-1.06)	0.88 (0.86-0.90)	0.93 (0.92-0.95)	1.03 (1.00-1.06)	0.94 (0.91-0.96)
HS diploma or less, %	1.09 (1.06-1.12)	1.14 (1.09-1.20)	0.95 (0.93-0.97)	0.97 (0.95-0.99)	1.03 (0.99-1.06)	0.91 (0.88-0.93)
Home ownership, %	0.89 (0.86-0.91)	1.08 (1.02-1.13)	1.02 (0.99-1.04)	1.05 (1.03-1.07)	1.04 (1.01-1.08)	0.98 (0.96-1.00)
Population per km^2^	0.94 (0.91-0.97)	0.94 (0.89-0.99)	1.18 (1.15-1.21)	0.98 (0.96-1.00)	0.91 (0.88-0.94)	1.07 (1.04-1.09)

Abbreviations: aOR, adjusted odds ratio; HS, high school; NH, non-Hispanic.

^a^ All linear variables are standardized such that estimates are equal to a 1-SD increase from the mean.

^b^ Models mutually adjusted for all change characteristics (2000-2010), as well as baseline (2000) living below poverty, HS diploma or less, and population density.

^c^ Racial/ethnic composition was assessed by predominant (>60%) racial/ethnic group. Places with no predominant group were classified as racially/ethnically mixed areas. For 2000 to 2010, census tracts that remained predominantly one race/ethnicity were grouped in their own category. Change in racial/ethnic composition was classified in the following 3 ways: (1) a change from predominantly NH white to predominantly NH black, predominantly Hispanic/Latino, predominantly NH Asian/Pacific Islander, or racially/ethnically mixed; (2) a change to predominantly NH white from predominantly NH black, predominantly Hispanic/Latino, predominantly NH Asian/Pacific Islander, or racially/ethnically mixed; and (3) all other changes. The reference group is remaining predominantly NH white.

^d^ Results for predominantly NH Asian/Pacific Islander census tracts that lost or had no health care facilities are suppressed because of small sample size (n <10).

Overall, decreases in neighborhood-level socioeconomic status were associated with never having or losing health care facilities (Table 4). Census tracts that had increases in the percentage of residents living below poverty level and having a HS diploma or less were less likely to gain health care facilities. However, CTs that had increases in the percentage of individuals living below poverty were also less likely to never have a health care facility. Results for change in home ownership were more mixed: an increase in the percentage of individuals who own homes was associated with higher odds of losing or gaining ambulatory care facilities and never having or losing pharmacies and drugstores (vs consistent presence), but an increase in the percentage of individuals who own homes was also associated with lower odds of never having (vs always having) ambulatory care facilities.

## Discussion

In this cross-sectional study of neighborhoods across the continental United States over a 15-year period, we found differential change in the presence of health care facilities across neighborhoods, with more socioeconomically disadvantaged neighborhoods never having or losing facilities. Specifically, we observed that CTs of areas with predominantly minority residents (NH black or Hispanic/Latino) or racially/ethnically mixed residents and CTs of areas with a higher percentage of residents living in poverty had a lower number of health care facilities compared with other neighborhoods. There is increasing evidence that racial/ethnic disparities in access to health care have been reduced for some subgroups after implementation of the Patient Protection and Affordable Care Act.^29,30,31^ However, our findings suggest that differential change in geographic presence of health care facilities by neighborhood demographic composition may further widen disparities in population health. Prior studies^32,33,34^ indicate that patterns of health care use among racial/ethnic minorities and low-income communities are associated with factors beyond geography, including physician-patient language concordance and health insurance constraints. We also observed increased likelihood of gaining health care facilities over time across all racial/ethnic composition categories, including those with changing racial/ethnic composition, while simultaneously observing decreased likelihood of gaining health care facilities over time among CTs of areas with increasing poverty and lower educational attainment. Complex characteristics and multilayered intricacies of racial/ethnic minority neighborhoods and ethnic enclaves can play beneficial and disadvantageous roles in geographic health care access and health outcomes.^35,36,37^ Furthermore, emerging data specifically on Asian American residential density suggest bimodal distributions in socioeconomic characteristics and other patterns that are unique from other racial/ethnic communities.^38^ More nuanced understanding of neighborhood racial/ethnic composition and more complex measurement are warranted, but more complex measurement was beyond the scope of this study. Therefore, increased research is needed on how the geographic presence of health care facilities and use of services are operationalized differently across population subgroups.

In addition, we observed that higher neighborhood socioeconomic status was associated with an increased number of health care facilities across neighborhoods. Medically underserved areas and populations are identified as geographic areas and populations with a lack of geographic access to primary care services.^39^ These areas are eligible for federal grants and health programs, such as Federally Qualified Health Centers, and are based on ratios of area-level population to health care providers, poverty level, percentage of population older than 65 years, and infant mortality rate.^39^ However, even after controlling for several of these factors, our findings suggest that areas with predominantly minority residents continue to disproportionately lack health care facilities. These trends over time may indicate a need for more targeted efforts to address disparities in access to ambulatory care services. Although prior studies^40,41,42,43,44,45^ have focused on geographic barriers to hospitals and tertiary care, few studies^3,46,47^ have examined trends in access to ambulatory care within neighborhoods across the nation; therefore, a critical understanding of these patterns is warranted.

### Strengths and Limitations

To our knowledge, this cross-sectional study is one of the first studies to examine longitudinal change in the presence of health care facilities across neighborhoods in the United States over a 15-year period. We focused on the presence of nonhospital facilities, thus giving a context for health care services that would provide primary care and care across the life span, and used detailed data on businesses for more accurate geographic location data and dates of operation of each facility.

However, some limitations should be noted. First, we focused on the presence of health care facilities within CTs and did not examine use of services among populations within CTs; therefore, we were unable to directly link availability with use. Second, the context of health care markets and concentrations of health care providers vary across the United States, and state and regional policies, market entry forces, and patterns of health care consolidation may have shaped the patterns observed^48^ but were beyond the scope of this study. Third, we used a broad classification of racial/ethnic composition at CTs, which limited our ability to examine more nuanced associations within and across population groups of high and low density. Fourth, we focused on 2 broad categories of health care facilities, including ambulatory care facilities (which encompass a wide variety of outpatient care services) and pharmacies and drugstores, to limit the consequences of misclassification; however, errors may have remained. Our study specifically spanned a period when urgent care clinics and retail pharmacies and drugstores were increasing, potentially accounting for the longitudinal change in specific areas.^49,50^ Limitations of the NETS data have also been noted in our group’s prior work.^51^

## Conclusions

Given the importance of geographic access to care on health outcomes, it is critical to monitor the spatial distribution of health care resources within the context of population health disparities. The Patient Protection and Affordable Care Act expanded overall health care access to primary care through insurance coverage, including Medicaid expansion in several states. However, even insured populations may face geographic barriers to accessing ambulatory care. Therefore, it remains important to understand neighborhood context and geographic access to health care resources when designing population health programs and policies.

## Notes

Supplement. **eTable 1.** Categorization of National Establishment Time Series (NETS) Data to Identify Health Care Facilities **eTable 2.** Description of Demographic and Socioeconomic Characteristics (2010) Across Categories of Change in Health Care Facilities (2000-2014) for Continental Nonwater US Census Tracts (n = 72246) **eFigure 1.** Proportion of Census Tracts in Each State Falling Within Each Ambulatory Care Category **eFigure 2.** Proportion of Census Tracts in Each State Falling Within Each Pharmacy Category **eFigure 3.** Proportion of Census Tracts Falling Within Each Ambulatory Care Category by Racial Composition **eFigure 4.** Proportion of Census Tracts Falling Within Each Pharmacy Category by Racial Composition
