# Supplementary material for: Patterns in Geographic Access to Health Care Facilities Across Neighborhoods in the United States Based on Data From the National Establishment Time-Series Between 2000 and 2014
Source: JAMA Netw Open. 2020 May 15;3(5):e205105. doi: 10.1001/jamanetworkopen.2020.5105 (PMC7229525; doi:10.1001/jamanetworkopen.2020.5105)
Supplement: Supplement. — eTable 1. Categorization of National Establishment Time Series (NETS) Data to Identify Health Care Facilities eTable 2. Description of Demographic and Socioeconomic Characteristics (2010) Across Categories of Change in Health Care Facilities (2000-2014) for Continental Nonwater US Census Tracts (n = 72246) eFigure 1. Proportion of Census Tracts in Each State Falling Within Each Ambulatory Care Category eFigure 2. Proportion of Census Tracts in Each State Falling Within Each Pharmacy Category eFigure 3. Proportion of Census Tracts Falling Within Each Ambulatory Care Category by Racial Composition eFigure 4. Proportion of Census Tracts Falling Within Each Pharmacy Category by Racial Composition [file jamanetwopen-3-e205105-s001.pdf]

Supplementary Online Content

Tsui J, Hirsch JA, Bayer FJ, et al. Patterns in geographic access to health care facilities across neighborhoods in the United States based on data from the National Establishment Time-Series between 2000 and 2014. *JAMA Netw Open*. 2020;3(5):e205105. doi:10.1001/jamanetworkopen.2020.5105

eTable 1. Categorization of National Establishment Time Series (NETS) Data to Identify Health Care Facilities

eTable 2. Description of Demographic and Socioeconomic Characteristics (2010) Across Categories of Change in Health Care Facilities (2000-2014) for Continental Nonwater US Census Tracts (n=72246)

eFigure 1. Proportion of Census Tracts in Each State Falling Within Each Ambulatory Care Category

eFigure 2. Proportion of Census Tracts in Each State Falling Within Each Pharmacy Category

eFigure 3. Proportion of Census Tracts Falling Within Each Ambulatory Care Category by Racial Composition

eFigure 4. Proportion of Census Tracts Falling Within Each Pharmacy Category by Racial Composition

This supplementary material has been provided by the authors to give readers additional information about their work.

© 2020 Tsui J et al. *JAMA Network Open*.

<table>
<tr><td colspan="3">eTable 1. Categorization of National Establishment Time Series (NETS) Data to Identify Health Care Facilities</td></tr>
<tr><th>Main Category for Analyses</th><th>NETS RECVD Category</th><th>SIC Code</th></tr>
<tr><td rowspan="2">Drug Stores and Pharmacies
Drug stores and pharmacies. Locations representing stores where medicine and/or drugs are dispensed or sold. May not include those that are also supermarkets. Includes pharmacies and drug stores defined by SIC code OR having a chain name on the TDLinx name list.</td><td>SIC-code based drug stores and pharmacies</td><td>SIC Code in the range 59120000-59129999 or SIC Code in 80110203</td></tr>
<tr><td>Name-search drug stores and pharmacies using TDLinx</td><td>SIC codes 53000000-53999999, 54000000-54999999, 56000000-56999999, 59000000-59999999, 65120200-65120299 AND Company or Trade Name on the TDLinx list for trade channel “Drug” and sub-channels “Conventional Drug Store” or “Rx Only & Small Drug Store.”</td></tr>
<tr><td rowspan="8">Ambulatory Care
Locations able to treat and provide chronic, continuous, outpatient care including screenings and other preventive measures. These places both allow an individual to maintain their health and address non-acute, non-emergency conditions</td><td>Mental Health Outpatient and continuous care</td><td>SIC Codes 80110400, 80110401, 80110402, 80110403, 80490400, 80490401, 80490403, 80490404, 80939902</td></tr>
<tr><td>Behavioral Health Outpatient and continuous care</td><td>SIC Codes 80930100,80930101,80930102,80930103</td></tr>
<tr><td>Urgent Care</td><td>SIC code 80110204</td></tr>
<tr><td>Retail clinics</td><td>SIC code 80990103, 80990201</td></tr>
<tr><td>Physical therapist</td><td>SIC code 80110521, 80490200, 80490201, 80939903</td></tr>
<tr><td>Kidney centers</td><td>SIC code 80920000</td></tr>
<tr><td>Offices or clinics of Health practitioners</td><td>SIC codes in the ranges 80110100-80110199, 80110500-80110514, 80119900-80119999, 80490100-80490199, 80930200-80930299, 80930300-80930399 or SIC codes 80110000, 80110202, 80110205, 80110517, 80110518, 80110519, 80110520, 80110522, 80110523, 80110524, 80310000, 80410000, 80420000, 80420100, 80420101, 80420102, 80420103, 80420105, 80429900, 80429901, 80430000, 80490000, 80499900, 80499902, 80499904, 80499906, 80499908, 80499909, 80930000, 80939900, 80939905, 80990200, 80999906, 80999907</td></tr>
<tr><td>Dental Care</td><td>SIC codes in the range 80210100-80210105 or SIC Codes 80210000, 80210107, 80210108, 80210200, 80210201, 80210202, 80219902</td></tr>
</table>

© 2020 Tsui J et al. *JAMA Network Open*.

**eTable 2.** Description of Demographic and Socioeconomic Characteristics (2010) Across Categories of Change in Health Care Facilities (2000-2014) for Continental Nonwater US Census Tracts (n=72246)									
		Ambulatory Care				Pharmacies and Drug Stores			
	All Tracts	None	Lose	Gain	Always	None	Lose	Gain	Always
N (tracts)	72246	6035	2020	10644	53547	32223	5650	11563	22810
Characteristic (2010)	% (n)	% (n)	% (n)	% (n)	% (n)	% (n)	% (n)	% (n)	% (n)
Racial Composition[a,c]									
Pred. White	63.9 (46158)	49.2 (2970)	48.9 (988)	63.6 (6764)	66.2 (35436)	62.9 (20253)	61.4 (3470)	64.6 (7464)	65.6 (14971)
Pred. Black	7.0 (5075)	14.9 (898)	18.5 (373)	6.6 (700)	5.8 (3104)	7.9 (2555)	9.8 (551)	5.3 (616)	5.9 (1353)
Pred. Hisp.	6.5 (4657)	10.6 (642)	9.2 (186)	7.2 (767)	5.7 (3062)	7.1 (2273)	5.9 (331)	6.0 (691)	6.0 (1362)
Pred. Asian	0.4 (262)	0.2 (9)	0.1 (1)	0.3 (32)	0.4 (220)	0.3 (97)	0.2 (13)	0.5 (55)	0.4 (97)
Mixed	22.3 (16094)	25.1 (1516)	23.4 (472)	22.4 (2381)	21.9 (11725)	21.9 (7045)	22.7 (1285)	23.7 (2737)	22.0 (5027)
	Mean (SD)	Mean (SD)	Mean (SD)	Mean (SD)	Mean (SD)	Mean (SD)	Mean (SD)	Mean (SD)	Mean (SD)
Non-US born, %[b]	12.1 (13.7)	10.2 (14.0)	10.2 (13.1)	11.2 (12.9)	12.6 (13.8)	11.3 (13.2)	11.2 (12.7)	13.7 (13.7)	12.6 (14.5)
Age >= 75, %[c]	6.3 (4.5)	5.0 (4.3)	5.6 (3.7)	5.2 (4.6)	6.7 (4.4)	5.8 (4.5)	6.5 (4.0)	5.9 (4.6)	7.3 (4.4)
Living Below Poverty, %[b]	15.8 (12.7)	20.8 (15.6)	22.4 (14.7)	14.4 (12.4)	15.4 (12.2)	15.9 (13.4)	17.5 (13.0)	13.4 (11.7)	16.7 (11.9)
HS diploma or less, %[b]	43.4 (18.5)	54.3 (18.7)	54.4 (16.0)	44.0 (18.1)	41.9 (17.9)	44.5 (18.9)	45.1 (17.6)	38.7 (17.9)	44.3 (17.5)
Home ownership, %[c]	63.8 (23.1)	62.0 (26.8)	61.6 (23.4)	71.1 (21.8)	63.0 (22.3)	66.5 (23.5)	61.3 (21.7)	66.5 (22.6)	60.1 (21.5)
Population per sq. km[c]	2017.5 (4533.1)	1788.1 (4185.2)	1948.5 (3864.9)	1495.2 (3635.6)	2149.8 (4741.6)	1785.1 (3820.3)	1887.1 (3623.4)	2017.2 (4334.4)	2378.4 (5610.9)

[a]Racial composition determined by prominent racial group (>60%). Places with no prominent group, classified as racially mixed areas.
[b]Data estimates from ACS 2008-2012.
[c]Data come from decennial census 2010.

© 2020 Tsui J et al. *JAMA Network Open*.

eFigure 1. Proportion of Census Tracts in Each State Falling Within Each Ambulatory Care Category

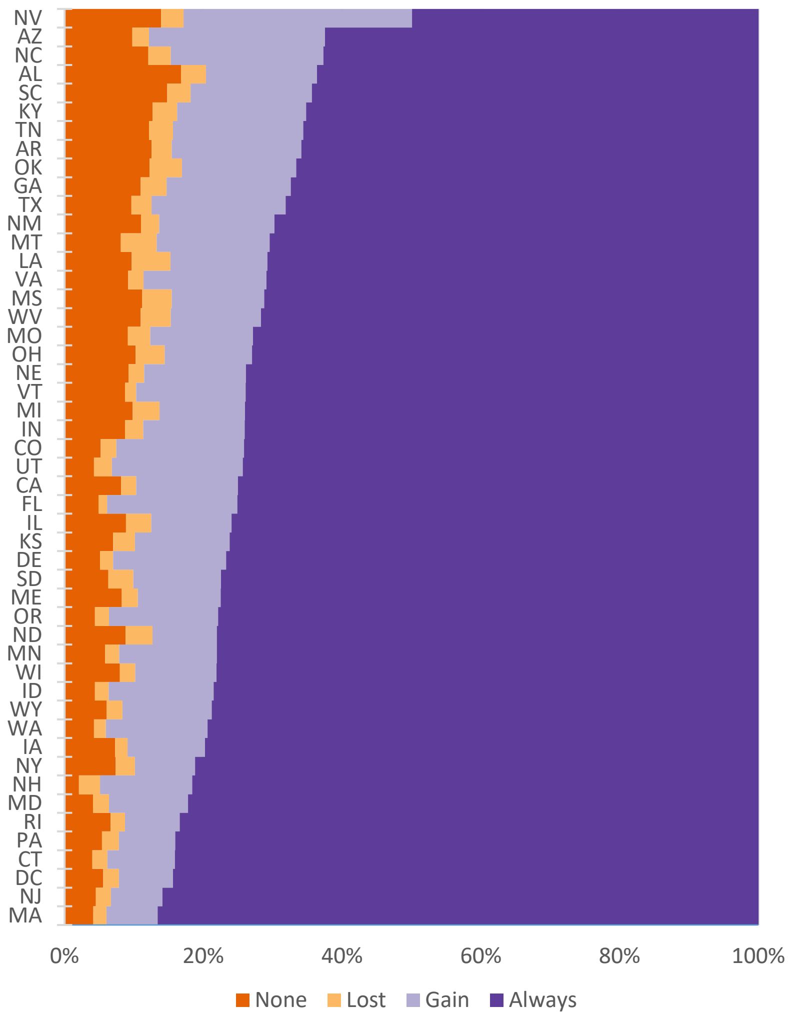

eFigure 2. Proportion of Census Tracts in Each State Falling Within Each Pharmacy Category

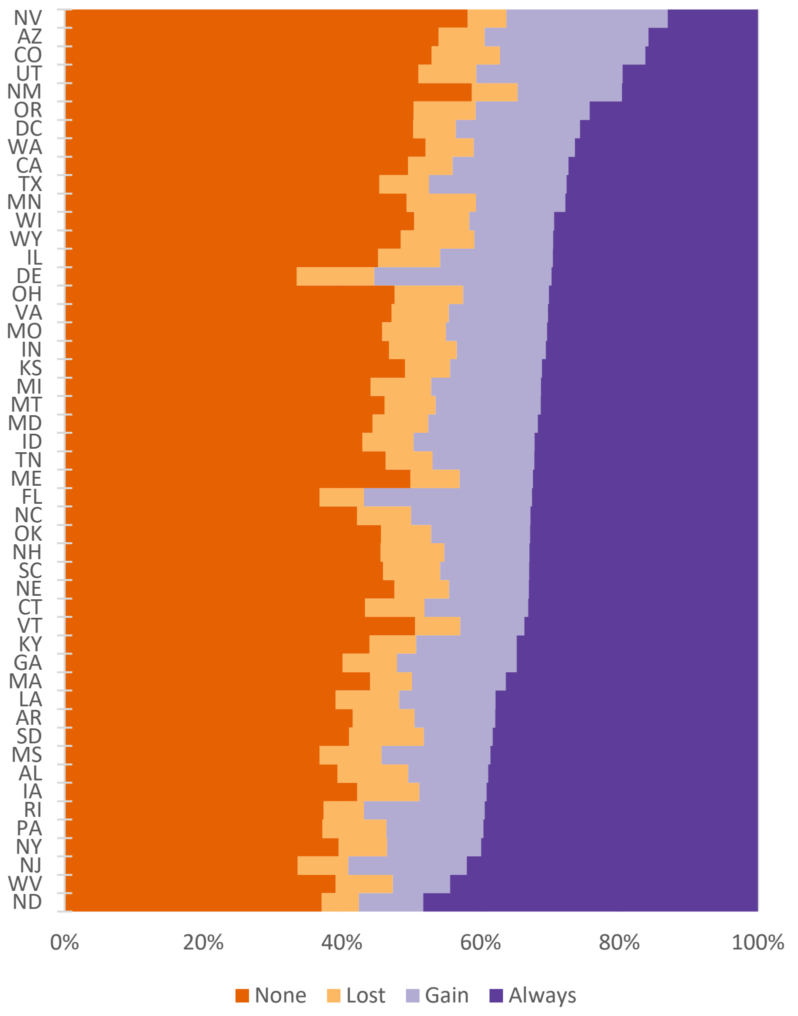

© 2020 Tsui J et al. *JAMA Network Open*.

eFigure 3. Proportion of Census Tracts Falling Within Each Ambulatory Care Category by Racial Composition

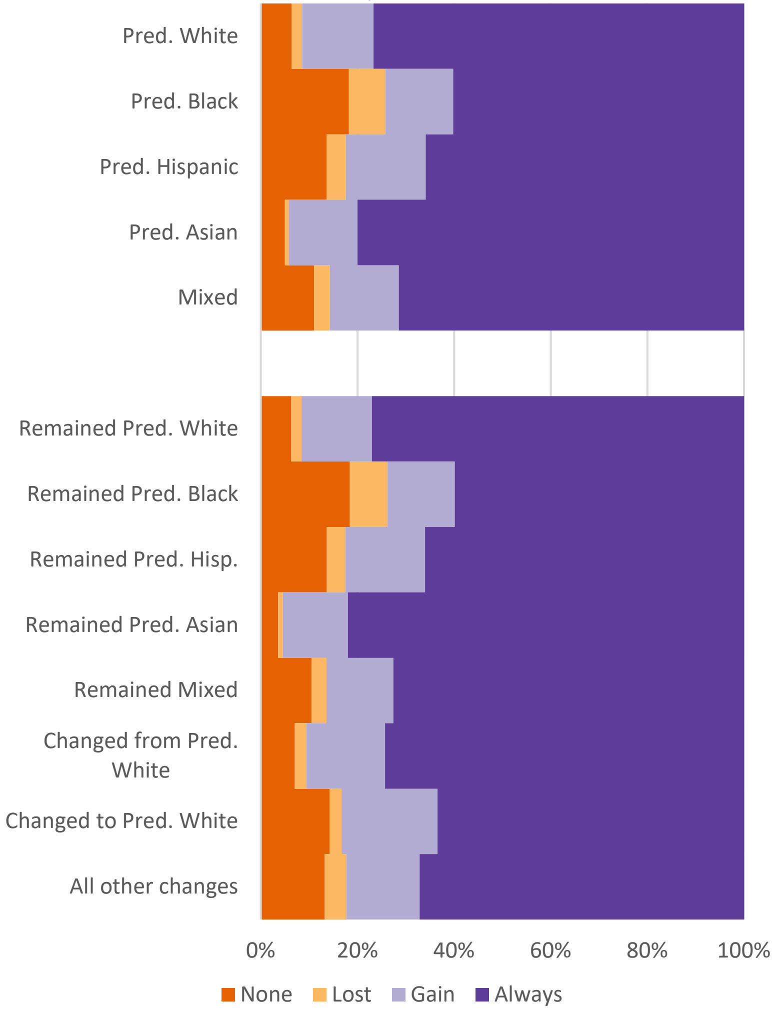

eFigure 4. Proportion of Census Tracts Falling Within Each Pharmacy Category by Racial Composition

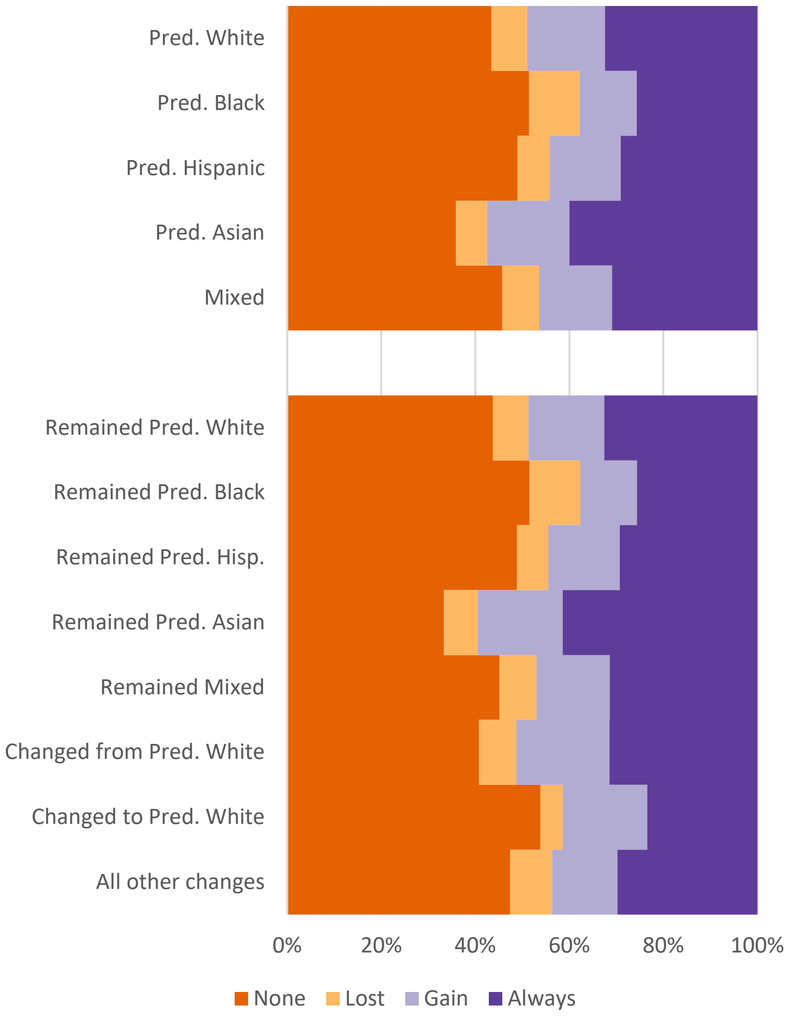

© 2020 Tsui J et al. *JAMA Network Open*.
